# The Influence of Negative Feedback and Social Rank on Feelings of Shame and Guilt: A Vignette Study in 8- to 13-Year-Old Non-Clinical Children

**DOI:** 10.1007/s10578-021-01143-4

**Published:** 2021-02-22

**Authors:** Eline Hendriks, Peter Muris, Cor Meesters

**Affiliations:** 1 Department of Clinical Psychological Science, Maastricht University, P.O. Box 616, 6200 MD Maastricht, The Netherlands; 2 Stellenbosch University, Stellenbosch, South Africa

**Keywords:** Self-conscious emotions, Shame and guilt, Negative feedback, Social rank, Children

## Abstract

This experimental study examined the role of negative feedback and social rank in the experience of self-conscious emotions, shame and guilt, in typically developing children aged 8 to 13 years. Participants were tested by means of a vignette paradigm in which feedback and social rank were systematically manipulated and levels of shame and guilt were assessed after listening to each of the vignettes. In addition, children completed a set of questionnaires for measuring individual differences in shame and guilt proneness, social comparison, submissive behavior, and external shame. The results showed that children presented with negative feedback reported higher ratings of shame and guilt than when presented with positive feedback, implying that the provision of negative feedback has a significant impact on children’s experience of self-conscious emotions. Social rank had less effect on children’s report of these self-conscious emotions. Furthermore, the individual difference variables of guilt proneness, and to a lesser extent shame proneness and submissive behavior, appeared to be positively related to self-conscious emotions as reported during the vignette task.

## Introduction

The self-conscious emotions of shame and guilt both involve negative self-evaluations elicited when some moral or social standard is violated which is noticed by other people [1]. Thus, these emotions occur if one has performed an act that is inconsistent with the current norms and values or one’s own expectations. This will cause disapproval by others hence leading to rejection or losing face. Self-conscious emotions are often labelled as ‘social emotions’ as they guide us through our busy social environments and help us to attain the key social goals of “getting along” and “getting ahead” [2]. Tracy and Robins [3] distinguish five unique features of self-conscious emotions as compared to basic emotions. First, different from basic emotions, self-conscious emotions always require self-awareness and self-representation. Second, self-conscious emotions are more complex as they require more sophisticated cognitive operations. Third, as a result these emotions emerge later in development than basic emotions. The fourth distinctive feature of self-conscious emotions is that they, unlike basic emotions, do not have discrete universally recognized facial expressions. Finally, self-conscious emotions serve to facilitate the attainment of complex social goals.

### Difference Between Shame and Guilt

While the distinction between self-conscious emotions and basic emotions is understood better nowadays, researchers still find it difficult to differentiate between the self-conscious emotions of shame and guilt. Both emotions are elicited by similar types of situations [4, 5] and for this reason the labels of shame and guilt are often used interchangeably. However, following the pioneering work by Lewis [6], evidence has accumulated indicating that these emotions are quite different. Lewis argued that guilt is typically characterized by a negative evaluation of a specific behavior (“I did *that* wrong”), whereas shame is more concerned with a negative evaluation of the global self (“*I* did that wrong”). More specifically, one who experiences guilt is feeling regret and remorse over the bad thing done, wishing that he/she would have behaved differently, and thinking about ways to undo the deed. In contrast, shame pertains to a negative evaluation of oneself as a person and is typically characterized by a sense of shrinking, feelings of worthlessness, and powerlessness. One feels exposed, which leads to a desire to escape or to disappear [1, 3]. Both emotions lead to different behavior in interpersonal contexts. More specifically, feelings of guilt motivate reparative behavior by making apologies and engaging in attempts to fix the situation, while feelings of shame prompt the person to show defensive and avoidance behavior [7].

### Development of Shame and Guilt

As noted earlier, self-conscious emotions are cognitively complex, and children need to develop at least three important cognitive skills in order to be able to express and experience emotions like guilt and shame [8]. First, children need to have a capacity of self-awareness and be able to form stable self-representations, which normally emerge between 18 and 24 months [9]. This means that they are able to differentiate between the self and the other, possess self-permanence, and consider the self as a separate entity. Second, children should become aware of the fact that there are particular rules and standards that define socially appropriate behavior. They have to evaluate their own behavior according to those standards and subsequently make attributions about the self [10]. Third, children must develop a theory of mind, which refers to the capability to attribute thoughts, feelings, and intentions to other persons [11]. Children come to realize that other people have expectations regarding their behavior and view them from an external, evaluative perspective. Due to further cognitive maturation, the external evaluations of children’s behavior are gradually internalized, which makes stable self-evaluations possible [12]. From the age of 8 onwards, most children experience feelings of shame and guilt and have a good comprehension of these emotions [2, 13, 14].

Shame and guilt are normal phenomena that are experienced by all children. These emotions are in essence functional as they facilitate and smoothen social interactions. However, there are clear individual differences in the degree to which a person reacts with shame and guilt, and both emotions can be dysfunctional if they are too intense or occur too frequently, or if they are very limited or hardly ever experienced. Dysregulation of shame and guilt is associated with a diversity of psychopathological outcomes, including anxiety, depression, and anger/aggression [2]. Within this article we focus on the question why some children experience higher levels of shame and guilt than others.

### Feedback

Muris and Meesters [2] assume that contextual factors play an important role in the emergence of individual differences in these self-conscious emotions. A first factor mentioned by these authors is concerned with receiving feedback, especially negative feedback. When people fail and violate their own or social values, other people alert them to this by giving feedback. Emotion research suggests that providing positive feedback will generally lead to positive emotions, such as happiness and pride, whereas negative feedback will generally result in negative emotions, including sadness but also the self-conscious emotions of shame and guilt [15]. In line with the above-described notions by Lewis [6], feedback will lead to shame when it is fueling a negative evaluation of the self. In contrast, if the negative feedback triggers a negative evaluation that is restricted to a specific action or behavior, feelings of guilt will occur [16, 17].

Direct feedback, provided by different people in various settings, can be both verbal and non-verbal in nature. Feedback given by parents helps the child to understand the meaning and significance of moral and social actions. Moreover, feedback contributes to a child’s internalization of standards for and expectations of acceptable behavior. Subsequently, children learn to evaluate and interpret their own experiences based on these rules. Research has shown that the negative valence of parental feedback contributes to more intense experiences of self-conscious emotions [18, 19]. In occupational situations, feedback has also been shown to be important. In a study by Belschak and Den Hartog [20], emotional reactions of employees were measured after they received performance feedback from their supervisors at work. It was found that feedback influenced the emotional reactions of employees. More specifically, those receiving positive feedback experienced more positive emotions, while those receiving negative feedback reported higher levels of negative emotions. Moreover, within a sample of adolescents, Belschak and Den Hartog [20] found that when negative feedback was received while other people were present, the negative emotions were experienced even more intensely. In another study by Buckley et al. [21], acceptance and rejection of a person in social interactions were also considered as a form of feedback. With social acceptance most people felt better about themselves and responded more socially, whereas rejection and feeling unvalued by others caused shame. People can also say things to belittle others and hurt others’ feelings, even when there is no specific failure. Shame and guilt have also been shown to increase by this type of negative feedback [22]. The levels of guilt in the receiver were even higher when disappointment was shown by the feedback provider [23]. Individuals have a need to belong and want to feel accepted by the people around them [21] and messages are emotionally hurtful if they imply a relational devaluation. Thus, rejection, exclusion, or negative feedback are likely to elicit self-conscious emotions. However, abovementioned studies are all conducted in adults and to the present authors’ knowledge so far no studies have been conducted to examine the relationship between negative feedback and shame and guilt in children.

### Social Rank

Another contextual factor that is thought to be implicated in the experience of guilt and shame has to do with the position in the social hierarchy or, briefly, social rank. According to the social rank theory, people have an innate need to be perceived by others as beings of high rank or status within a group [24]. This desire is already present in early childhood and the perception of one’s own social rank strongly influences one’s emotions and moods. Thus, if a child, for some reason, has the impression that he/she is inferior to others, this determines the way he/she deals with social encounters. A low social rank, which is involuntary and unwanted, leads to an increased tendency to behave submissively. Submissive behavior can consist of eye gaze avoidance, fear grinning, backing down, and a lack of confidence causing one to make no claims on resources or efforts to advertise oneself. Consequently, submissive behavior is often accompanied by self-conscious emotions like shame and guilt [25].

Based on social rank theory, one would expect that a person with a higher social status would be less prone to experience shame or guilt than a person with a lower social status. However, research on the effect of social rank on self-conscious emotions in children is sparse, and the only study conducted so far has yielded quite unexpected results. Stapleton et al. [14] compared the anticipated shame and guilt experiences of high and low social status children. A group of children, consisting of 74 boys and 89 girls aged 8 to 12 years, were asked to nominate classmates as popular, socially preferred, respected, and overtly aggressive. Next, they were exposed to vignettes in which the protagonist was confronted with a number of shame- and guilt-eliciting events, and instructed to indicate how they would feel and act in these situations. Results indicated that only children who were popular and high in social rank indicated that they would experience shame and guilt in response to the hypothetical transgressions. In children who were perceived as popular but not particularly high in social rank and children with a low social status, no significant effects on self-conscious emotions were documented. Altogether, these findings are not in agreement with the social rank theory and suggest that the relation between social status and the emotions of shame and guilt might be rather complicated. However, it should be noted that Stapleton et al. [14] used peer nomination to measure the social rank of children instead of asking the children themselves how they experienced their position within the group of peers. As indicated earlier, experiencing self-conscious emotions is related to one’s self-image. Therefore, it would be better to manipulate children’s perception of their position within the social group, which could be effectively done by means of a vignette paradigm [26].

### Present Study

To summarize, findings suggest that feedback, and negative feedback in particular, plays a more prominent role in the experience of shame and guilt than social rank. However, previous studies have been mainly conducted with adults and used a variety of methods, which may not provide a fair comparison between both contextual factors. With this in mind, the present study was conducted to further examine the effects of negative feedback and social rank on the experience of the self-conscious emotions of shame and guilt in children. A vignette paradigm was employed to directly manipulate feedback and social rank in a sample of non-clinical children aged 8 to 13 years. Children listened to scenarios in which negative feedback versus positive feedback, and high social rank versus low social rank were systematically varied. Following each scenario, children were asked to rate how much guilt and shame they would experience in the described situation. With regard to feedback, we hypothesized that scripts in which negative feedback was given would yield higher levels of self-conscious emotions than scripts in which positive feedback was provided. With respect to social rank, predictions were less clear but on the basis of social rank theory, one might expect that scripts which prompted a low social rank would elicit higher levels of self-conscious emotions than scripts which prompted a high social rank.

We also administered self-report questionnaires that assessed a number of constructs that were considered relevant for understanding the inter-individual variations in the experience of shame and guilt. Specifically, children completed the child version of the Test of Self-Conscious Affect (TOSCA; [27]) as a measure of habitual shame and guilt proneness. The Adolescent Social Comparison Scale (ASCS; [25]) was used as an index of self-perceived social qualities in comparison with others (which may provide a proxy index of children’s social rank). Furthermore, the children completed the Adolescent Submissive Behavior Scale (ASBS; [25]) and other as Shamer Scale (OAS; [28]), which assess individual differences in submissive behavior and external shame (i.e., the distressing notion that others evaluate the person in a negative way), respectively. It was anticipated that higher levels of dispositional shame and guilt (TOSCA-C), lower ratings of social comparison (ASCS), and higher levels of submissiveness (ASBS) and external shame (OAS) would be associated with higher shame and guilt ratings in response to the vignettes. Children with disproportionate high levels of shame and guilt have been shown to be more prone to develop psychopathology [29]. It seems valuable to gain better insights into contextual variables that play a role in the development of these self-conscious emotions. This type of information could create greater awareness in parents and teachers about the way they provide feedback to children and the importance of children’s position in social groups. If research could demonstrate that these factors significantly contribute to children’s negative self-conscious emotions, they could be appropriate targets in both prevention and early intervention programs.

## Method

### Participants and Procedure

Children were recruited from seven primary schools in the south-eastern part of the Netherlands. All children in the three oldest age groups were invited to participate by sending their parents a letter, which provided background information about the study, along with a consent form. Eventually, the parents of 103 children (58 boys and 45 girls) responded positively to this invitation, which means that the participation rate was 28%. Children had a mean age of 10.76 years (*SD* = 0.94, range 8–13 years) and, with the exception of one child, all of them were from original Dutch descent. Children completed the set of questionnaires and audiotaped vignette paradigm (see below). This was either done individually in a separate room in the school in the presence of a research assistant or online by use of Microsoft Teams, which was a procedure that was implemented during the Covid-19 pandemic.

### Measures

#### Vignette Paradigm

A vignette paradigm contains stories by which researchers try to elicit responses that reveal attitudes, beliefs, social norms, and perceptions of the participant [30]. For the purpose of this study, six vignettes were designed to measure the effects of positive/negative feedback and high/low social rank on feelings of shame and guilt. Vignettes described normal events that might occur in the life of children of this age, namely: inviting children to your birthday party, playing with friends, losing a game, working together in school, making something dirty, and having a minor bicycle accident. For each of the six situations, four different scripts were made in which either the type of feedback towards the child (positive versus negative) or the social rank of the child (high versus low) was systematically manipulated (see for an example: Table 1). The total of 24 scripts, divided in four blocks, were audiotaped to ensure that all participants received the information in a similar way. Scripts were presented in a random order with two exceptions: (a) Each block of six scripts contained scripts of all six situations; and (b) Scripts containing the same manipulation never followed one after another (e.g., two scripts with positive feedback were not presented in a row). To minimize carry-over effects, the order of the scripts was reversed (counterbalanced) for half of the children. After each script, two statements were provided: (1) “You feel guilty, because you did something wrong,” and (2) “You are ashamed and feel bad about yourself.” The child rated these two statements on a 5-point Likert scale with 1 being “absolutely not true” and 5 being “very true.” Children were instructed to listen carefully to all scripts; they were told in advance that the scripts would be very similar, but that there were also small differences among them.

**Table 1:** Example of a vignette: Invitation to a birthday party

Negative feedback	It is your birthday soon and you are throwing a party. During the break, all children are standing around you when you hand out the invitations. You give one classmate no invitation to your birthday party. *The classmate without an invitation is very disappointed and walks away sadly*
Positive feedback	It is your birthday soon and you are throwing a party. During the break, all children are standing around you when you hand out the invitations. You give one classmate no invitation to your birthday party. *The classmate without an invitation smiles at you*
Low social rank	It is your birthday soon and you are throwing a party. *You do not have any real friends, but you decide to invite some kids anyway*. During the break, all children are standing around you when you hand out the invitations. You give one classmate no invitation to your birthday party
High social rank	It is your birthday soon and you are throwing a party. *Almost everyone in your class is your friend and you want to invite everyone*. During the break, all children are standing around you when you hand out the invitations. You give one classmate no invitation to your birthday party

#### Questionnaires

The Dutch version of the *Test of Self-Conscious Affect for Children* (TOSCA-C; [27]) is a questionnaire that elicits negative self-conscious emotions and thoughts [31]. The TOSCA-C contains 15 brief scenarios that young people are likely to encounter in day-to-day life: 10 scenarios have a negative content (e.g., failing a test, forgetting to buy a birthday present for your mother, or spilling someone else’s drink) and 5 scenarios have a positive content (e.g., your report card shows you are one of the best students in the class, the teacher picks you to do something special). Each scenario is followed by a number of associated responses. Children are asked to imagine themselves in the given situation and to think about how they would feel and think. Next, they are asked to rate each response on a 5-point scale (with 1 = ‘very untrue’ and 5 = ‘very true’) to indicate the likelihood of reacting in the manner indicated. Two responses following each scenario pertain to the experience of shame and guilt in that situation. Shame items describe self-labelling (e.g., feelings of incompetency), affect (e.g., feelings of disgust), and escape or avoidance behavior, whereas guilt items reflect feelings for the other person, making amends, and thoughts about doing things a better way [24]. Besides items of shame and guilt proneness, other TOSCA-C items measure externalization, detachment or unconcern, alpha pride, and beta pride [25] but these subscales were not used in the present study. The internal consistency of the shame and guilt proneness subscales is good (α = 0.77 and α = 0.81, respectively), and their validity has also been supported [32]. Finally, it has been noted that the shame and guilt proneness scores are substantially positively correlated in child and adolescent samples [33].

A Dutch translation and adaptation of the *Adolescent Social Comparison Scale* (ASCS; [24]) was used to measure children’s self-perceived social qualities as compared to others. Young respondents have to indicate how they compare themselves with peers in terms of shyness, smartness, popularity, differentness, attractiveness, strength, acceptance, quietness, confidence, and loneliness. For each of the 10 ASCS items (e.g., “In relation to your friend, how *shy* do you feel?”), children rate their answer on a 10-point Likert scale (1 = ‘feeling much less than peers’, and 10 = ‘feeling much more than peers’). A total score can be computed by summing the responses to all items, after reversing the scores on the negatively phrased items of shyness, differentness, quietness, and loneliness. Thus, a higher total score represents a more favorable social comparison (i.e., high-scoring participants feel they function socially better than their peers). This measure was used in several studies and has shown to have a Cronbach’s α of around 0.90 [13, 24].

The *Adolescent Submissive Behavior Scale* (ASBS; [25]) is an adapted version of the Submissive Behavior Scale for adults [34]. The Dutch translation of this questionnaire contains 12 items that measure the degree of submissive behavior. Children are asked to rate how they would behave in certain conflictual situations with their peers. The scale includes items such as: “When I am with people of my own year, I do things because others are doing them, rather than because I want to.” Answers are given on a 5-point Likert scale, ranging from 1 = ‘never’ to 5 = ‘always’ [24]. A total score can be calculated by summing scores across all items. Higher scores indicate higher levels of submissive behavior [35]. The scale has been used in a number of previous studies [24, 25] and evidence has been obtained for its reliability (e.g., Cronbach’s α = 0.85) and validity [36].

A Dutch translation and adaptation of the *Other as Shamer Scale* (OAS [28]); was used to measure ‘external shame’, or, in other words, how negative children think other people will see them. The OAS consists of 18 items (e.g., “I feel other people see me as not good enough,” or “I feel insecure about other opinions of me”), that are rated on a 5-point Likert scale, ranging from 0 = ‘never’ to 4 = ‘almost always.’ A total score can be calculated by summing the scores on individual items, with a higher score indicating a higher level of external shame. Previous research has demonstrated that the OAS is a valid measure and that its internal consistency is good (α = 0.92) [37].

### Statistical Analyses

The Statistical Package for Social Sciences (SPSS, Version 26) was used to compute descriptive and reliability statistics of various questionnaires, to conduct paired samples *t*-tests in order to compare guilt and shame ratings in response to various types of vignettes (i.e., high versus low social rank and negative versus positive feedback), and to examine unique associations among study variables by means of correlations and regression analyses.

## Results

### General Findings

Before addressing the main results of the study, a number of general findings will be discussed (see Table 2). First, normality tests were conducted for all scales used in this study. For the vast majority of the variables, a normal distribution of scores was found. In fact, the only exception was the ASBS, for which a skewness of 1.11 and kurtosis of 3.60 was documented. Because additional analyses with log transformed ASBS data yielded highly similar findings, it was decided to report the results of the analyses without adjustments. Second, a number of statistically significant gender differences were found. As expected, girls scored higher on the shame proneness subscale of the TOSCA-C [*t*(101) = 2.02, *p* < 0.05, *d* = 0.40] as compared to boys. Further, girls also displayed higher shame scores in response to the vignettes, irrespective of their precise content in terms of feedback or social rank [all *t*(101)’s ≥ 2.49, *p*’s < 0.05, *d*’s ≥ 0.49). Girls also displayed higher guilt scores in response to both low and high social rank vignettes [*t*(101)’s being 2.43, *p* < 0.05, *d* = 0.49 and 2.24, *p* < 0.05, *d* = 0.44, respectively] than did boys. Third, guilt proneness as measured by the TOSCA-C was significantly negatively associated with age (*r* = − 0.20, *p* < 0.05), which implies that with increasing age dispositional guilt scores tended to decrease. No other age effects were found, but it should be noted that the age range of the children in this sample was rather small. Fourth, all questionnaires proved to be reliable in this sample of non-clinical children, with Cronbach’s alphas varying between 0.67 and 0.89. Vignette paradigm scores also displayed acceptable reliability, with Cronbach’s alphas ranging between 0.64 and 0.75. Fifth and finally, correlations among the questionnaires used in this study showed the to-be-expected pattern (see Table 3). For example, shame proneness was significantly positively related to guilt proneness (*r* = 0.60, *p* < 0.001), submissive behavior (*r* = 0.45, *p* < 0.001), and external shame (*r* = 0.46, *p* < 0.001), and significantly negatively related to social comparison (*r* = − 0.33, *p* < 0.01), whereas guilt proneness was significantly positively related to submissive behavior (*r* = 0.31, *p* < 0.01). Further, submissive behavior was significantly positively related to external shame (*r* = 0.53, *p* < 0.001), and significantly negatively related to social comparison (*r* = − 0.29, *p* < 0.01). And finally, social comparison was significantly negatively related to external shame (*r* = − 0.31, *p* < 0.01).

**Table 2:** Descriptive statistics (means, standard deviations, gender differences, and internal consistency coefficients) of various questionnaires included in the present study

	Total sample (*N* = 103)	Boys (*n* = 58)	Girls (*n* = 45)	Cronbach’s α
TOSCA-C				
Shame proneness	41.06 (8.80)	39.53 (8.73)	43.02 (8.60)*	0.80
Guilt proneness	53.76 (7.26)	53.00 (7.60)	54.73 (6.76)	0.74
ASBS submissive behavior	30.91 (6.42)	31.09 (6.75)	30.69 (6.03)	0.68
ASCS social comparison	62.02 (11.39)	62.48 (11.38)	61.42 (11.51)	0.67
OAS external shame	16.77 (10.72)	16.21 (10.07)	17.49 (11.57)	0.89
Vignette paradigm				
Shame—Negative feedback	19.54 (4.55)	18.59 (4.16)	20.78 (4.77)*	0.71
Shame—Positive feedback	14.63 (4.11)	13.45 (3.74)	16.16 (4.10)***	0.67
Guilt—Negative feedback	21.21 (4.05)	20.55 (3.92)	22.07 (4.10)	0.68
Guilt—Positive feedback	15.79 (4.38)	15.29 (4.53)	16.42 (4.13)	0.71
Shame—High social rank	17.90 (4.66)	16.45 (4.12)	19.78 (4.68)***	0.75
Shame—Low social rank	18.56 (4.42)	17.40 (4.16)	20.07 (4.33)**	0.68
Guilt—High social rank	19.96 (4.04)	19.19 (3.93)	20.96 (4.01)*	0.66
Guilt—Low social rank	20.00 (4.12)	19.16 (4.29)	21.09 (3.64)*	0.64

*ASBS* Adolescent submissive behavior scale, *ASCS* Adolescent social comparison scale, *OAS* Other as shamer scale, *TOSCA-C* Test of self-conscious affect for children

Asterisks indicate gender differences: *p < 0.05; **p < 0.01; ***p < 0.001

**Table 3:** Correlation coefficients between the questionnaires included in the present study

	1	2	3	4	5
1. TOSCA-C Shame proneness					
2. TOSCA-C Guilt proneness	0.60***				
3. ASBS Submissive behavior	0.45***	0.31**			
4. ASCS Social comparison	− 0.33**	− 0.15	− 0.29**		
5. OAS External shame	0.46***	0.13	0.53***	− 0.31**	

*ASBS* Adolescent submissive behavior scale, *ASCS* Adolescent social comparison scale, *OAS* Other as shamer scale, *TOSCA-C* Test of self-conscious affect for children *p < 0.05; **p < 0.01; ***p < 0.001

### Feedback and Self-Conscious Emotions

The effects of negative versus positive feedback on children’s shame and guilt ratings during the vignette task were examined by means of paired samples *t*-tests. The results showed that children exposed to vignettes in which the protagonist received negative feedback reported higher levels of shame (*M* = 19.54, *SD* = 4.55) than children exposed to vignettes in which positive feedback was given (*M* = 14.63, *SD* = 4.11) [*t*(102) = 12.70, *p* < 0.001, *d* = 1.13]. Similar results were documented for feelings of guilt: children reported higher levels of this self-conscious emotion in response to negative feedback vignettes (*M* = 21.21, *SD* = 4.05) than in response to positive feedback vignettes (*M* = 15.79, *SD* = 4.38) [*t*(102) = 12.89, *p* < 0.001, *d* = 1.29]. Note that in both analyses the effect sizes associated with the feedback manipulation were large, which indicates that this contextual factor had a substantial impact on children’s experience of these self-conscious emotions. Results of additional *t*-tests indicated that independent of the precise feedback information of the vignettes, children displayed higher levels of guilt than levels of shame [*t*(102)’s being 5.56, *p* < 0.001, *d* = 0.39 and 4.21, *p* < 0.001, *d* = 0.27, respectively]. In other words, children in general reported more guilt than shame in response to feedback-related vignettes.

### Social Rank and Self-Conscious Emotions

The effects of the social rank (high versus low) manipulation on the reported self-conscious emotions of shame and guilt during the vignette task were examined by means of paired samples *t*-tests. There was a statistically significant difference in shame scores between low (*M* = 18.56, *SD* = 4.42) and high (*M* = 17.90, *SD* = 4.66) social rank vignette conditions [*t*(102) = 2.25, *p* < 0.05, *d* = 0.15]. Meanwhile, the social rank manipulation did not produce a differential effect on children’s guilt scores [*t*(102) = 0.13, *p* = 0.90]. The effect size for the difference in children’s shame ratings between the high and low social rank conditions was small. Results of additional *t*-tests again indicated that children in general reported higher levels of guilt than shame in response to both low and high social rank vignettes [*t*(102)’s being 5.28, *p* < 0.001, *d* = 0.34 and 7.77, *p* < 0.001, *d* = 0.47, respectively]. Altogether, these results indicate that social rank, as manipulated by the content of the vignettes, had some impact on children’s experience of shame but no influence on the experience of guilt.

### Individual Difference Variables and Self-Conscious Emotions

Partial correlations (controlled for gender) between children’s shame and guilt ratings in response to various types of vignettes and the questionnaires that were included to assess a number of relevant constructs are shown in Table 4. TOSCA-C shame and guilt proneness scales were significantly and positively correlated with shame and guilt ratings as obtained during the vignette task, and these correlations were fairly independent of the precise content of the vignettes in terms of feedback or social rank (all *r*’s between 0.20 and 0.50, all *p*’s < 0.05). ASBS scores showed similar positive correlations with children’s shame and guilt ratings in response to the vignettes (all *r*’s between 0.20 and 0.37, all *p*’s < 0.05). Thus, higher levels of submissive behavior were associated with higher levels of these self-conscious emotions. Findings with regard to social comparison as measured by the ASCS were less consistent. Only in response to negative feedback vignettes, a lower level of self-perceived social functioning as compared to others was associated with higher levels of shame and guilt (*r*’s = − 0.20 and − 0.21 respectively, both *p*’s < 0.05). Further, ASCS scores also correlated significantly negatively with guilt ratings in response to low social rank vignettes (*r* = − 0.22, *p* < 0.05), implying that children who felt they functioned socially better than their peers reported lower levels of guilt in the low social rank condition. No significant correlations were found between the external shame as indexed by the OAS and guilt and shame ratings in response to various types of vignettes.

**Table 4:** Correlations (corrected for gender) among the vignette paradigm and other questionnaires

	TOSCA-C Shame proneness	TOSCA-C Guilt Proneness	ASBS Submissive behavior	ASCS Social comparison	OAS External Shame
Feedback					
Shame—Negative feedback	0.37***	0.48***	0.26**	− 0.20*	0.08
Shame—Positive feedback	0.31**	0.35***	0.20*	0.09	0.03
Guilt—Negative feedback	0.28**	0.46***	0.24*	− 0.21*	0.03
Guilt—Positive feedback	0.20*	0.33***	0.27**	0.07	0.04
Social rank					
Shame—Low social rank	0.47***	0.47***	0.33***	− 0.15	0.11
Shame—High social rank	0.49***	0.49***	0.30**	− 0.08	0.04
Guilt—Low social rank	0.36***	0.50***	0.37***	− 0.22*	0.15
Guilt—High social rank	0.34***	0.45***	0.35***	0.05	0.05

*ASBS* Adolescent submissive behavior scale, *ASCS* Adolescent social comparison scale, *OAS* Other as shamer scale, *TOSCA-C* Test of self-conscious affect for children

*p < 0.05; **p < 0.01; ***p < 0.001

### Unique Links Between Individual Differences Constructs and Self-Conscious Emotions

Given the strong intercorrelations among the individual differences constructs as well as the finding that most of them correlated in a theoretically meaningful way with children’s shame and guilt ratings in response to the vignettes, regression analyses were carried out to explore their unique contributions to reported self-conscious emotions during the vignettes task. A hierarchical approach was chosen in which the (significantly correlated) individual differences measures were all simultaneously added to the model (on step 2) after controlling for gender (on step 1). Table 5 summarizes the main results of these analyses. In the interest of space, we only show (a) the models for which the predictors explained a statistically significant proportion of the variance in the dependent variables, and (b) the statistics for the significantly contributing variables.

**Table 5:** Main results of the regression analyses predicting children’s reported feelings of shame and guilt in various conditions of the vignette paradigm

	B	SE	*β*	ΔR^2^
Shame—Negative feedback				0.24***
TOSCA-C Guilt proneness	0.25	0.07	0.40***	
Guilt—Negative feedback				0.23***
TOSCA-C Guilt proneness	0.25	0.06	0.45***	
Shame—Positive feedback				0.13***
TOSCA-C Guilt proneness	0.14	0.06	0.24*	
Guilt—Positive feedback				0.14**
TOSCA-C Guilt proneness	0.19	0.07	0.32**	
ASBS Submissive behavior	0.15	0.07	0.21*	
Shame—Low social rank				0.27***
TOSCA-C Guilt proneness	0.17	0.06	0.27**	
TOSCA-C Shame proneness	0.12	0.06	0.24*	
Guilt—Low social rank				0.29***
TOSCA-C Guilt proneness	0.24	0.06	0.42***	
ASBS Submissive behavior	0.13	0.06	0.21*	
Shame—High social rank				0.27***
TOSCA-C Guilt proneness	0.19	0.06	0.29**	
TOSCA-C Shame proneness	0.14	0.06	0.26*	
Guilt—High social rank				0.24***
TOSCA-C Guilt proneness	0.20	0.06	0.37***	
ASBS Submissive behavior	0.14	0.06	0.22*	

*ASBS* Adolescent submissive behavior scale, *TOSCA-C* Test of self-conscious affect for children

*p < 0.05; **p < 0.01; ***p < 0.001

First of all, it was found that gender made a significant contribution to most models with the exception of the guilt ratings in response to negative and positive feedback vignettes, accounting for 5 to 13% of the variance in shame and guilt scores. Second and most importantly, individual differences variables explained 13 to 29% of the variance in children’s shame and guilt ratings during various conditions of the vignette task. Of these individual differences variables, TOSCA-C guilt proneness made a unique and significant contribution to every model, regardless of the predicted self-conscious emotion (shame or guilt) or the type of vignette (i.e., negative or positive feedback, or high or low social rank). Further, shame proneness accounted for an independent and significant proportion in the variance of children’s shame scores in low and high social rank vignettes (*β*’s = 0.24 and 0.26 respectively, *p*’s < 0.05). Finally, ASBS submissive behavior was found to explain unique variance in guilt scores in response to positive feedback vignettes (*β* = 0.21, *p* < 0.05*)* and to low and high social rank vignettes (*β*’s = 0.21 and 0.22 respectively, *p*’s < 0.05).

## Discussion

The present study adopted a vignette approach to examine the role of two contextual factors (i.e., feedback and social rank) to the experience of feelings of shame and guilt in a non-clinical population of children aged 8–13 years. The results suggest that feedback is an important factor in children’s experience of self-conscious emotions. More precisely, it was found that children reported substantially higher levels of both shame and guilt after listening to vignettes in which the protagonist received negative feedback as compared to vignettes in which positive feedback was given. Furthermore, social rank was found to have much less impact on children’s report of these self-conscious emotions. That is, in response to vignettes in which the protagonist had a low social rank, children reported slightly but still significantly higher levels of shame than in response to scenarios in which the protagonist had a high social rank, while no statistically significant effect of social rank on children’s reports of feelings of guilt was observed.

Receiving negative feedback can be considered as an emotionally salient event. Moreover, when feedback is given in public rather than in private, stronger negative emotional reactions, including shame and guilt, can be expected [20, 38]. The vignettes that were used in the present study indeed described situations in which negative verbal feedback was given in the presence of peers, and this may have resulted in the rather strong effects on children’s reports of shame and guilt. Although the results of our study regarding the experience of self-conscious emotions following feedback were straightforward and as anticipated, it needs to be emphasized that in real-life the relation between this contextual factor and experiences of shame and guilt is far more complex. For instance, there are clear indications that the way the feedback is provided is more important than the content of the feedback or the person who delivers it. Evidence for this comes from a study by Johnson and Connelly [23] who found that in particular displays of disappointment when giving negative feedback had the potential to elicit self-conscious emotions in the recipient of the critical comments.

The effects of social rank on children’s reports of self-conscious emotions were not particularly convincing. In line with social rank theory [7, 24, 39], low social rank descriptions in the vignettes elicited somewhat higher levels of shame than high social rank descriptions, whereas no such an effect could be noted for the self-conscious emotion of guilt. The most likely explanation for this finding is that children’s perception of social rank may be rather difficult to manipulate by means of vignettes. More precisely, the impact of negative feedback is to some extent universal, as it is never nice to hear that your moral or social behavior is not in line with norms and expectations. As a result, it may have been relatively easy for children to identify with the protagonist in the vignettes and hence report on the associated self-conscious emotions. In contrast, the social rank manipulation was rather indirect: for example, a child had to infer high versus low social rank from the fact that he/she had many or few friends, which might be more indicative of sociability than of actual social rank. Moreover, the effect of the social rank manipulation may critically depend on a child’s perceived position within the peer group. In other words, it may be rather difficult for a child with a high social rank to imagine themselves in a low social rank position and vice versa for a child with a low position to picture themselves in a high social rank, which is one potential reason why the reports on associated self-conscious emotions did not show the predicted pattern.

In the present study, we included a number of relevant individual difference variables, including shame and guilt proneness, submissive behavior, social comparison, and external shame. Of these variables, shame and guilt proneness (TOSCA-C) and submissive behavior (ASBS), were found to be the most consistent and robust correlates of shame and guilt as reported by the children during the vignette task. In all cases, correlations were positive, indicating that children who were dispositionally more susceptible to display shame and guilt responses and those who had a greater inclination to exhibit submissive behavior were more likely to report higher levels of both self-conscious emotions during the vignette paradigm. Social comparison (ASCS) and external shame (OAS) appeared to be less convincingly correlated with children’s reports of shame and guilt during the vignette task. This could indicate that these constructs were less relevant within a context of self-conscious emotions, but it might also be that these adolescent measures were less suitable and hence less valid for children at this younger age (i.e., 8 to 13 years).

Additional regression analyses carried out to explore the unique contributions of individual difference variables to children’s shame and guilt scores in response to the vignettes revealed that guilt proneness (TOSCA-C) was a particularly important correlate. In all significant models, guilt proneness emerged as a significant and unique positive predictor of self-conscious emotions—either shame or guilt—as reported by children during the vignette task. In a number of models, shame proneness and submissive behavior were also noted to have an independent and statistically significant positive impact. The dominance of guilt proneness may well have to do with the fact that this was a non-clinical population in which guilt scores were in general more often reported as compared to shame. It is possible that in clinically referred children, shame proneness will become more relevant as previous studies have indicated that shame is the more ‘pathological’ form of self-conscious emotion [2, 40], but obviously this remains a point of future inquiry.

A final result of the present study that is worth mentioning pertains to the gender differences that were noted in self-conscious emotions. In line with what has been documented in the literature [41], it was found that girls in general displayed statistically significant higher levels of shame and guilt as compared to boys, and this was not only true for shame proneness as measured by the TOSCA-C but also for most of the children’s shame and guilt scores as obtained during the vignette task. Thus, it can be concluded that girls seem to be more susceptible to experience these self-conscious emotions than boys.

## Limitations and Future Research

The current study was one of the first that examined the role of the contextual variables of feedback and social rank on the experience of the self-conscious emotions of shame and guilt in children. It should be acknowledged, however, that this investigation suffered from various limitations. First, for this study, a vignette paradigm was used in order to study the effects of (negative versus positive) feedback and (high versus low) social rank. Vignettes have been criticized due to their limited ecological validity [42]. As such, by asking children to imagine and respond to a number of hypothetical social situations, it cannot be assured that the results represent children’s actual (emotional) reactions to the described events. A second drawback was that we did not have exact information on children’s actual social rank. Although we included a measure of social comparison, this scale can only be seen as a proxy of perceived social rank, and as discussed earlier it may well be that children’s actual social status may have had an important influence on reports of self-conscious emotions, in particular to high and low social rank vignettes. A third shortcoming concerns the cross-sectional nature of our study. Although we relied on an experimental manipulation to elicit self-conscious emotion in children by varying the relevant contextual factors of feedback and social rank, the correlations between individual difference variables and children’s reports of shame and guilt in response to the vignettes cannot be interpreted in terms of cause-effect relations. The fourth and final limitation is related to the use of a non-clinical sample, which means that the findings cannot be generalized to clinically referred children.

Given the abovementioned limitations, future research should make an attempt to study children’s shame and guilt reactions to feedback and social rank manipulations in experimental set-ups mimicking real-life events (e.g., giving an oral performance, conducting a difficult task with a group of children). Further, prospective studies should be conducted to assess the temporal associations between shame and guilt, relevant individual differences variables, and symptoms of psychopathology in children. In this way, the interplay between contextual variables and self-conscious emotions as well as their role in the development of psychological problems could be examined. Lastly, it seems also important to conduct this type of research in clinical settings and to find out whether feedback and social rank have a similar impact on the experience of shame and guilt of children who already suffer from emotional problems.

Obviously, it is good that children receive feedback from caregivers as this promotes awareness of what is socially and morally accepted and corrects deviant and faulty behavior. The emotions of shame and guilt are guiding during this process as they make children realize that something has gone wrong and prompt them to undertake corrective action. Meanwhile, it is important to keep in mind that the way feedback is given should not be too negative: harsh, denigrating, and publicly visible commentary on children’s faulty actions could promote a strong sense of self-consciousness that is associated with too intense feelings of shame and guilt, which will hinder children to develop a healthy sense of sociality and morality. In the psychological literature, example of studies can be found showing associations among parental psychopathology, negative parenting styles, and self-conscious emotions in children [43, 44]. It would be worthwhile to focus on the role of negative feedback in these relations, and to target this factor in prevention and early intervention programs for at-risk families. Teachers at school should also think about the manner they provide feedback to children. It is already known that positive feedback contributes to a good atmosphere in class that facilitates children’s learning process [45], but inevitably teachers sometimes also have to give negative feedback. They need to be aware that feedback in a too negative way can have a significant impact on children’s emotional development.

## Summary

While previous studies on the role of feedback and social rank on self-conscious emotions primarily focused on adolescents or adults, this is one of the first investigations on this topic that was conducted in children. Our results indicate that in particular negative feedback promotes feelings of shame and guilt in children, while the role of social rank seems to be less important. Furthermore, shame and guilt proneness and submissive behavior were found to be individual differences that influence a child’s experience of self-conscious emotions. The present study was subject to a number of limitations, which also provide leads for future research on this topic. Further inquiry on the role of feedback and social rank in the development of shame and guilt is important because this type of research could inform parents, teachers, and other caregivers on how to frame these environmental factors in such a way that children develop into socially and morally well-functioning human beings that do not suffer from high levels of self-consciousness and psychological problems.
